# The Patient Assessment of Chronic Illness Care produces measurements along a single dimension: results from a Mokken analysis

**DOI:** 10.1186/s12955-017-0638-4

**Published:** 2017-04-04

**Authors:** C. J. Gibbons, N. Small, J. Rick, J. Burt, M. Hann, P. Bower

**Affiliations:** 1 Cambridge Centre for Health Services Research, University of Cambridge, Cambridge, UK; 2 The Psychometrics Centre, University of Cambridge, Cambridge, UK; 3 Centre for Primary Care, University of Manchester, Manchester, UK

## Abstract

**Background:**

As the worldwide prevalence of chronic illness increases so too does the demand for novel treatments to improve chronic illness care. Quantifying improvement in chronic illness care from the patient perspective relies on the use of validated patient-reported outcome measures. In this analysis we examine the psychometric and scaling properties of the Patient Assessment of Chronic Illness Care (PACIC) questionnaire for use in the United Kingdom by applying scale data to the non-parametric Mokken double monotonicity model.

**Methods:**

Data from 1849 patients with long-term conditions in the UK who completed the 20-item PACIC were analysed using Mokken analysis. A three-stage analysis examined the questionnaire’s scalability, monotonicity and item ordering. An automated item selection procedure was used to assess the factor structure of the scale. Analysis was conducted in an ‘evaluation’ dataset (*n* = 956) and results were confirmed using an independent ‘validation’ (*n* = 890) dataset.

**Results:**

Automated item selection procedures suggested that the 20 items represented a single underlying trait representing “patient assessment of chronic illness care”: this contrasts with the multiple domains originally proposed. Six items violated invariant item ordering and were removed. The final 13-item scale had no further issues in either the evaluation or validation samples, including excellent scalability (Ho = .50) and reliability (Rho = .88).

**Conclusions:**

Following some modification, the 13-items of the PACIC were successfully fitted to the non-parametric Mokken model. These items have psychometrically robust and produce a single ordinal summary score. This score will be useful for clinicians or researchers to assess the quality of chronic illness care from the patient's perspective.

## Background

Improving the quality of care for long-term conditions including arthritis, diabetes and coronary heart disease is a global healthcare priority. The increasing prevalence of multimorbidity (the co-existence of multiple long-term conditions in the same individual) adds additional pressures to individuals and healthcare systems alike [1].

The Patient Assessment of Chronic Illness Care (PACIC) is a relatively brief 20-item questionnaire designed to assess the extent to which care is aligned with the Chronic Care Model [2, 3]. The chronic care model (CCM) has been widely accepted as a suitable framework for improving the care of patients with long-term (‘chronic’) conditions such as diabetes or arthritis.

The PACIC has been widely used in both validation studies and as an endpoint in outcomes research [4–7]. A short version for cardiovascular disease patients has been developed using factor analysis [8, 9] but despite the scale’s popularity, no analysis has been performed using modern test theories, including either parametric and non-parametric item response theory [10].

Previous studies using confirmatory factor analyses failed to find support for the hypothesised 5-factor structure of the PACIC [9, 11] though other studies using exploratory factoring methods found better support for the original structure [12]. Disparities in findings related to the factorial structure leaves some uncertainty as to how the scale may be best applied to measure a patient’s assessment of their own care. The current study addresses this uncertainty be examining the scaling structure of the PACIC using modern psychometric methods [13], avoiding some of the known issues with illusory factors in factor analyses, which may be driving the uncertainty about the scale’s structure in the literature [14].

The current study conducted a psychometric analysis of the PACIC scale using Mokken analysis. Mokken analysis is analogous to non-parametric item response theory, and may be used to arrange ordinal questionnaire items into scales and to assess if the assumptions of non-parametric item response theory (including unidimensionality and monotonicity) are met by the scale (4). By successfully applying data to the Mokken model the suitability using ordinal scale sum scores is confirmed (Table 1).

**Table 1:** Details of the PACIC 20-item scale

PACIC 20 item was developed to measure patient perceptions of five elements seen as necessary for the succesful implementation of the chronic care model:	
Patient Activation (items 1–3)	
Delivery System Design/Decision Support (items 4–6)	
Goal Setting (items 7–11)	
Problem-solving/Contextual Counseling (items 12–15)	
Follow-up/Coordination (items 16–20)	

## Methods

Data for the analyses described here were originally collected as part of a wider cohort study designed to assess the impact of care planning on patient outcomes [7]. The current analyses use the baseline data from the cohort study. The same sample has previously been used to investigate the factor structure of PACIC and is described elsewhere [11]. Ethical approval was granted for the original data collection by Northwest 3 REC – Liverpool East (REC Ref no: 10/H1002/41).

Analyses in the current paper were all conducted within R Statistical Computing Environment [15] using the ‘base’ and ‘mokken’ packages [16, 17].

### Mokken analysis

Mokken models are a non-parametric extension of the simple deterministic Guttman scaling model [18]. Guttman models unrealistically assume that data are error free and Mokken models introduce a probabilistic framework which allows researchers to account for measurement error [19]. The major advantage of employing a non-parametric item response theory (NIRT) technique over other modern test theories, including the Rasch models [20], is the relatively relaxed assumptions within NIRT [21] whilst affirming important psychometric assumptions of unidimensionality and scalability [19].

Two Mokken models of interest are the monotone homogeneity model (MH model) and the double monotonicity model (DM model). In the MH model, items are allowed to differ in their discrimination parameter (the slope of their item characteristic curve). The DM model is a more restrictive version of the MH model where item discrimination parameters are fixed, much in the same way as the Rasch or 1 parameter item response theory (IRT) model. Within the MH model it is possible that some items have a weaker or stronger relationship than others to the underlying trait, which may indicate redundancy [19]. Fitting the DM model is essential in order to ensure that scores for polytomous questionnaires are correctly ordered [22].

Following suggestions in Mokken analysis teaching papers [16, 23] a three-stage analysis was conducted. These three stages of analysis ensure that four assumptions of NIRT are met. Both the assumptions of NIRT and the stages of a Mokken analysis are described below.

## Assumptions of non-parametric item response theory

### Unidimensionality

The assumption of unidimensionality states that all items must measure the same underlying latent trait. This assumption can be expressed both logically (that all items measure one construct) as well as mathematically (that only one latent variable is necessary to account for the inter-item associations within the data) [21].

### Local independence of items

The assumption of local independence simply states that an individual’s response to an item is reliant solely on their level of the underlying trait being measured and not influenced by their responses to other items on the same questionnaire.

Local dependence may occur where item content is too conceptually similar between items meaning that the response to one item is conditional on the response to another.

However, whilst sophisticated methods for assessing local independence of items have been reported and used under parametric IRT paradigms [24, 25], tests to assess local dependency under the NIRT paradigm are not, as far as the authors are aware, yet widely available in accessible psychometric packages [26].

### Monotonicity

The assumption of monotonicity states that the probability of affirming an item is a non-decreasing functioning of the level of the underlying latent trait. For example, on a given item a person with a high level of the underlying trait (theta) must always have a greater chance of affirming an item than a person with a lesser level of the underlying trait.

### Non-intersection

**Fig. 1:**
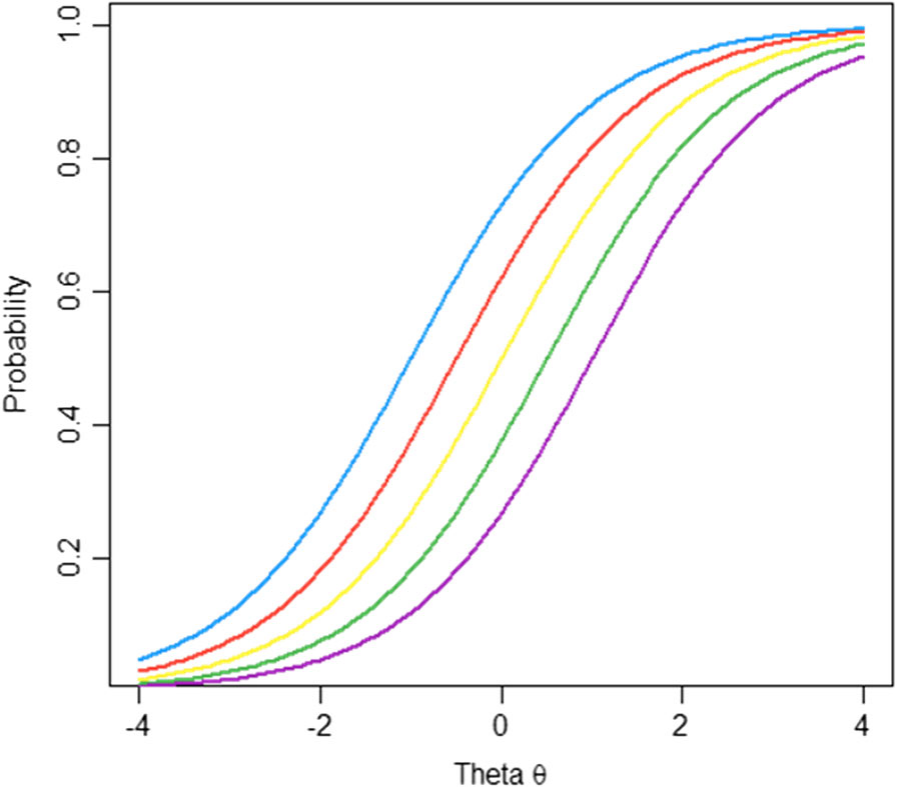
Non-intersecting item characteristic curves

**Fig. 2:**
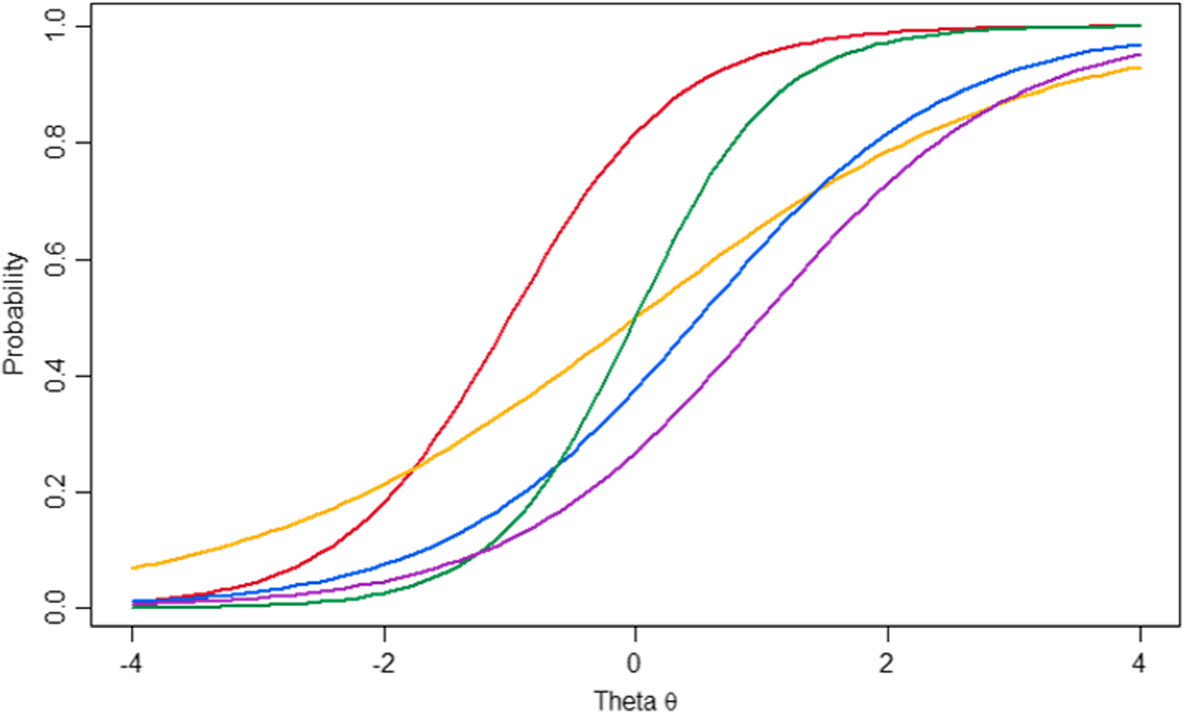
Intersecting item characteristic curves

An additional assumption of non-intersection is added in order to satisfy the demands of the more restrictive DM model. Non-intersection is confirmed by invariant item ordering which ensures that the ordering of each item (in terms of its ‘difficulty’) is the same for each individual responding to the scale. Invariant item ordering (IIO) occurs when the item characteristic curves intersect across the scale, which may not occur where slope parameters are uniform across the scale. Figure 1 gives an example of non-intersecting item characteristic curves and Fig. 2 shows item characteristic curves that intersect.

## Stages of Mokken analysis

### Stage one

In Stage One the scalability of both the individual item and scale total is evaluated using Loevinger’s H coefficient, where a higher value indicates higher scalability. The Mokken *‘*automated item selection procedure’ is also used at this stage to assess the number and structure of meaningful factors within the data.

Mokken (1971) suggested several ‘rules of thumb’ for assessing Loevinger’s scalability coefficients. A scale is considered weak if .3 ≤ *H* < .4, considered ‘moderately scalable’ if .4 ≤ *H* < .5 and strong if *H* ≥ .5.

This stage of a Mokken analysis is analogous to an exploratory factor analysis [17].

### Stage two

In Stage Two the assumption of monotonicity (higher scores indicate a high level of the trait or characteristic being measured) between item pairs within the sample is assessed. The ‘mokken’ package evaluates the number and severity of monotonicity violations. Items that violate the assumption of monotonicity should be removed to improve the scale.

### Stage three

The final assumption of invariant item ordering is to check for non-intersection using the *manifest invariant item ordering* protocol in the ‘mokken’ package. Invariant item ordering occurs when the ordering of the items is the same for each participant [27]. Items that violate this assumption may be removed from the scale one at a time following an iterative process. In the event that two items violate the assumption, the item with the lowest scalability is removed, before analysing the rest of the items again.

After the completion of all three stages, the final scale can be said to demonstrably meet all of the assumptions of non-parametric item response theory.

### Local independence

As no formal test of local independence exists under the Mokken NIRT paradigm the final items of the PACIC will be analysed for local independence by conceptual comparison of wording and item themes. Local independence may also be indirectly indicated by Loevinger’s H and Rho values that are exceptionally high.

### Reliability

Scale reliability will be calculated using the Molenaar Sijtsma statistic (Rho) [28]. The Rho statistic calculates the probability of obtaining the same score twice by extrapolating on the basis of the proportion of respondents who give positive responses to item pairs [13].

### Evaluation and validation sampling

To ensure that the findings in the current study would be robust across multiple different samples the sample was split randomly into an evaluation and validation sample. The analysis described above was then first run on the evaluation sample and confirmed by application to the validation sample.

## Results

### Data

The 1849 cases were split randomly into evaluation (*n* = 956) and validation (*n* = 890) samples.

#### Stage one

The Mokken automated item selection procedure (AISP) indicated that a single meaningful factor was present, which included all of the items within the dataset. Scalability coefficients (Item H) are given in Table 2. In its 20 item form, the scale displayed an acceptable overall H value of .50 (SE = .01).

**Table 2:** Item Scalability Coefficients for the PACIC scale

Item code	Item Wording	Item H
PACIC1	Asked for my ideas when we made a treatment plan.	.50
PACIC2	Given choices about treatment to think about.	.49
PACIC3^a^	Asked to talk about any problems with my medicines or their effects.	.48
PACIC4	Given a written list of things I should do to improve my health.	.49
PACIC5	Satisfied that my care was well organised.	.44
PACIC6	Shown how what I did to care of myself influenced my condition.	.49
PACIC7	Asked to talk about my goals in caring for my condition.	.54
PACIC8	Helped to set specific goals to improve my eating or exercise.	.52
PACIC9	Given a copy of my treatment plan.	.54
PACIC10^a^	Encouraged to go to a specific group or class to help me cope with my chronic condition.	.43
PACIC11	Asked questions, either directly or on a survey, about my health habits.	.49
PACIC12	Sure that my doctor or nurse thought about my values, beliefs, and traditions when they recommended treatments to me.	.50
PACIC13^a^	Helped to make a treatment plan that I could carry out in my daily life.	.55
PACIC14^a^	Helped to plan ahead so I could take care of my conditions even in hard times.	.57
PACIC15^a^	Asked how my chronic condition affects my life.	.54
PACIC16	Contacted after a visit to see how things were going.	.50
PACIC17	Encouraged to attend programmes in the community that could help me.	.48
PACIC18^a^	Referred to a dietitian, health educator, or counsellor.	.41
PACIC19^a^	Told how my visits with other types of doctors, like an eye doctor or other specialist, helped my treatment.	.44
PACIC20	Asked how my visits with other doctors were going.	.48

^a^Item removed from final analysis

### Stage two

Tests of monotonicity returned no violation of monotonicity for any item (see Tables 3 and 4).

**Table 3:** Summary of monotonicity

	Item H	Standard Error	Active comparisons	Violations
PACIC1	.50	.02	106	0
PACIC2	.49	.02	105	0
PACIC3	.48	.02	112	0
PACIC4	.49	.02	98	0
PACIC5	.44	.02	94	0
PACIC6	.49	.02	112	0
PACIC7	.54	.02	98	0
PACIC8	.52	.02	106	0
PACIC9	.54	.02	98	0
PACIC10	.43	.02	98	0
PACIC11	.49	.02	99	0
PACIC12	.50	.02	105	0
PACIC13	.55	.02	128	0
PACIC14	.57	.02	98	0
PACIC15	.54	.02	98	0
PACIC16	.50	.02	94	0
PACIC17	.48	.02	86	0
PACIC18	.41	.02	112	0
PACIC19	.44	.02	112	0
PACIC20	.48	.02	101	0

**Table 4:** Backwards step-wise removal of items violating IIO

	Step 1	Step 2	Step 3	Step 4	Step 5	Step 6	Step 7	Step 8
PACIC1	4	3	2	1	1	0	0	0
PACIC2	2	2	1	0	0	0	0	0
PACIC3	2	2	2	2	NA	NA	NA	NA
PACIC4	2	1	0	0	0	0	0	0
PACIC5	0	0	0	0	0	0	0	0
PACIC6	0	0	0	0	0	0	0	0
PACIC7	1	1	1	1	1	1	1	0
PACIC8	1	1	1	1	0	0	0	0
PACIC9	0	0	0	0	0	0	0	0
PACIC10	1	1	1	1	1	1	NA	NA
PACIC11	2	2	1	0	0	0	0	0
PACIC12	0	0	0	0	0	0	0	0
PACIC13	6	5	NA	NA	NA	NA	NA	NA
PACIC14	5	4	4	NA	NA	NA	NA	NA
PACIC15	2	2	2	2	1	1	1	NA
PACIC16	1	0	0	0	0	0	0	0
PACIC17	0	0	0	0	0	0	0	0
PACIC18	6	NA	NA	NA	NA	NA	NA	NA
PACIC19	3	3	2	1	1	NA	NA	NA
PACIC20	2	1	1	1	1	1	0	0

NA indicates that item was removed from the scale. Numbers represent the number of unidimensional scales present in the dataset, starting at 0 and rising in positive integers

Note: item numbers are based on the original order in which they were listed in the PACIC.

### Stage three

Assessment of IIO suggested that the 20-item scale did not have IIO properties and a process of backwards step-wise deletion was conducted, iteratively removing seven items over eight steps, illustrated in Table 5.

**Table 5:** Loevinger’s H coefficients for the final scale

	Item wording	Evaluation sample		Validation sample	
		Item H	se	Item H	se
PACIC1	Asked for my ideas when we made a treatment plan.	.48	.02	.51	.02
PACIC2	Given choices about treatment to think about.	.48	.02	.49	.02
PACIC4	Given a written list of things I should do to improve my health.	.48	.02	.50	.02
PACIC5	Satisfied that my care was well organised.	.45	.02	.47	.02
PACIC6	Shown how what I did to care of myself influenced my condition.	.52	.02	.52	.02
PACIC7	Asked to talk about my goals in caring for my condition.	.56	.02	.57	.02
PACIC8	Helped to set specific goals to improve my eating or exercise.	.53	.02	.54	.02
PACIC9	Given a copy of my treatment plan	.46	.03	.55	.02
PACIC11	Asked questions, either directly or on a survey, about my health habits.	.47	.02	.49	.02
PACIC12	Sure that my doctor or nurse thought about my values, beliefs, and traditions when they recommended treatments to me.	.50	.02	.50	.02
PACIC16	Contacted after a visit to see how things were going.	.47	.02	.50	.02
PACIC17	Encouraged to attend programmes in the community that could help me.	.41	.03	.45	.03
PACIC20	Asked how my visits with other doctors were going.	.41	.02	.46	.02

The removed items (3,10, 13,14,15,18 and 19) were originally formed part of the ‘Patient Activation’ (Item 3), ‘Goal Setting’ (item 10), ‘Problem Solving’ (Items 13–15) and ‘Follow-up’ (Items 18 and 19) domains.

The final “patient assessment of chronic illness care” scale consisted of 13-items that fully met all NIRT assumptions of dimensionality, scalability, monotonicity and invariant item ordering. The final scale H was .48 (SE = .01) indicating very good scalability.

### Validation analysis

To confirm the findings in the evaluation analysis the final 13-item scale was assessed in the validation half of the original dataset. The final 13 items solution demonstrated good scalability, monotonicity and did not violate the IIO assumption.

#### Reliability

The Molenaar Sijtsma statistic (Rho) indicated very good reliability in the final 13-item scale (Rho = .88).

## Discussion

Non-parametric Mokken analysis indicated that the items of the PACIC questionnaire a single unidimensional trait representing patient’s assessment of their chronic illness care, rather than the previously hypothesised five-factor structure. Within this single dimension, the 20 items of the PACIC displayed good scalability and monotonicity, however seven items displayed invariant item ordering; violating an assumption of the double monotonicity model. Upon removing these 6 items the resultant 13-item questionnaire displayed excellent scalability and reliability across a single dimension.

Three of the six items which were removed from the analysis were originally placed in the ‘Problem Solving’ domain (Items 13, 14 and 15), two from the ‘Follow-up’ domain (Items 18 and 19), one from the ‘Goal Setting’ (Item 10) and one from the ‘Patient Activation’ domains (Item 3). The removal of these items may relate to inconsistencies in the implementation of different elements of the CCM in the United Kingdom. Items 18 and 19 both assess activities carried out by other medical practitioners, these items appear to rely on the assumption that seeing another medical professions (e.g., dietician) is appropriate for all respondents.

Whilst these items remain in the questionnaire, the maximum score could not be attained from any patients with a chronic condition who did need to see other clinical staff such as a medical educator or ‘eye doctor’, which may have caused undue bias between patients who require care from multiple professionals and those who do not.

It is important that items which are meant to assess satisfaction with aspects of healthcare that may not be universally implemented are worded carefully to reduce confusion and facilitate accurate measurement [29].

We recommend that researchers and clinicians who wish to measure the views of patients relating to the quality of their chronic illness care in the UK are best to do so using the 13-item solution presented here, rather than the original scale across five dimensions for which we found no support in the current study. The scale has the advantage of being shorter, thus being less burdensome.

The present study is limited insofar as it was not possible to assess local independence of items using the tools available. Local dependency can result in inflated covariance between items which may, in turn, lead to higher H-coefficients and the risk that items with local dependency are spuriously included in the scale. However, in the absence of a quantitative analysis, some confidence can be gained from assessing the item wording for items which have clear conceptual overlap. It appears that the final 13 items do cover a broad range of topics and do nerlying trait ot have obvious conceptual overlap: which would be indicative of local dependency.

Further research may usefully be conducted on this scale that assesses the PACIC using parametric item-response theory, which may include other analyses including local independence of items and differential item functioning [29]. Parametric item-response theory also leads to the possibility of employing computer adaptive testing, which can improve the efficiency and accuracy of assessments [30].

The current study was conducted exclusively in the United Kingdom and significant heterogeneity in the way in which chronic care is organised and experienced globally suggests that the final 13-item solution may not hold for populations in the United States of America, for example. Another study which used factor analyses to assess the psychometric performance of the PACIC for use in diabetic populations in the USA using factor analyses reduced the number of items in the final scale to 11, the disparity between findings may be attributable to differing experiences of patients in the UK and the USA [10]. Given these differences, the recommendations made in this paper should not be applied to the PACIC when it is deployed within a US population for which it was originally developed. Work which derived a set of items which functioned well across populations would be tremendously useful to establish to enable comparison of global models of chronic healthcare from the patient perspective.

## Conclusions

The original PACIC scale was found to be unidimensional and, following the process of Mokken analysis, 13 items met the assumptions of scalability and unidimensionality, which are necessary for producing reliable, ordinal measurements from questionnaire scales. The removal of superfluous items that do not contribute positively to accurate unidimensional measurement has produced a 13-item version of the PACIC, which we recommend for use in the UK.

## Abbreviations

AISP: Automated item selection procedure
CCM: Chronic Care Model
DM: Double monotonicity
IIO: Invariant item ordering
IRT: Item response theory
MH: Monotone homogeneity
NIRT: Non-parametric item response theory
PACIC: Patient Assessment of Chronic Illness Care
SE: Standard error
